# Safety profile of HIV-1 attachment inhibitor prodrug BMS-663068 in antiretroviral-experienced subjects: week 24 analysis

**DOI:** 10.7448/IAS.17.4.19530

**Published:** 2014-11-02

**Authors:** Jacob Lalezari, Gulam H Latiff, Cynthia Brinson, Juan Echevarria, Sandra Treviño-Pérez, Johannes R Bogner, David Stock, Samit R Joshi, George J Hanna, Max Lataillade

**Affiliations:** 1 Quest Clinical Research, N/A, San Francisco, CA, USA; 2 Maxwell Clinic, N/A, Durban, South Africa; 3 Family Medicine, Southwestern Medical School, Austin Branch and Central Texas Clinical Research, Austin, TX, USA; 4 Hospital Nacional Cayetano Heredia, Lima, Peru; 5 HIV Research Department, Mexico Centre for Clinical Research, Mexico City, Mexico; 6 Section for Infectious Diseases, Med. IV, Hospital of the University of Munich, Munich, Germany; 7 Research and Development, Bristol-Myers Squibb, Wallingford, CT, USA; 8 Research and Development, Bristol-Myers Squibb, Princeton, NJ, USA

## Abstract

**Introduction:**

BMS-663068 is a prodrug of BMS-626529, an attachment inhibitor that binds directly to HIV-1 gp120, preventing initial viral attachment and entry into the host CD4+ T-cell. AI438011 is an ongoing, Phase IIb, randomized, active-controlled trial investigating the safety, efficacy and dose–response of BMS-663068 vs. atazanavir/ritonavir (ATV/r) in treatment-experienced (TE), HIV-1-positive subjects. At Week 24, response rates across the BMS-663068 arms were consistent with ATV/r.

**Materials and Methods:**

Antiretroviral TE subjects (exposure to ≥1 antiretroviral for ≥1 week) with susceptibility to all study drugs (including BMS-626529 IC_50_ 100 nM) were randomized equally to four BMS-663068 arms (400 or 800 mg, BID; 600 or 1200 mg, QD) and a control arm (ATV/r 300/100 mg QD), with tenofovir disoproxil fumarate (TDF) + raltegravir (RAL). The complete safety profile through Week 24 is reported.

**Results:**

In total, 251 subjects were treated (BMS-663068, 200; ATV/r, 51). No BMS-663068-related adverse events (AEs) led to discontinuation. Grade 2–4 drug-related AEs occurred in 17/200 (8.5%) subjects across the BMS-633068 arms; however, these events were mostly single instances and no dose-relationship was seen. Similarly, no noticeable trend for Grade 3–4 laboratory abnormalities was seen and Grade 3–4 hematologic changes and liver chemistry elevations were uncommon (neutropenia, 2.5%; AST/ALT elevations, 1% (*n*=196)). In the ATV/r arm, Grade 2–4 drug-related AEs occurred in 14/51 (27.5%) subjects and were mostly secondary to gastrointestinal and/or hepatobiliary disorders. Serious adverse events (SAEs) occurred in 13/200 (6.5%) and 5/51 (9.8%) subjects receiving BMS-663068 and ATV/r, respectively; most were secondary to infections and none were related to study drugs. The most common AE reported for BMS-663068 was headache (28/200, 14%), occurring in 5/51 (10%) subjects in the ATV/r arm; in the BMS-663068 arms, this was not dose-related. There were no deaths.

**Conclusions:**

BMS-663068 was generally well tolerated across all arms, with no related SAEs or AEs leading to discontinuation and no dose-related safety signals. There were no trends for Grade 2–4 AEs or clinical laboratory abnormalities. These results support continued development of BMS-663068.

**Note:**

Previously submitted at IDWeek, Philadelphia, PA, 8 October 2014.

